# Deep Convolutional Generative Adversarial Network-Based EMG Data Enhancement for Hand Motion Classification

**DOI:** 10.3389/fbioe.2022.909653

**Published:** 2022-07-29

**Authors:** Zihan Chen, Yaojia Qian, Yuxi Wang, Yinfeng Fang

**Affiliations:** College of Telecommunication, Hangzhou Dianzi University, Hangzhou, China

**Keywords:** EMG, DCGAN, data enhancement, inter-class distance, classification accuracy, histogram equalization

## Abstract

The acquisition of bio-signal from the human body requires a strict experimental setup and ethical approvements, which leads to limited data for the training of classifiers in the era of big data. It will change the situation if synthetic data can be generated based on real data. This article proposes such a kind of multiple channel electromyography (EMG) data enhancement method using a deep convolutional generative adversarial network (DCGAN). The generation procedure is as follows: First, the multiple channels of EMG signals within sliding windows are converted to grayscale images through matrix transformation, normalization, and histogram equalization. Second, the grayscale images of each class are used to train DCGAN so that synthetic grayscale images of each class can be generated with the input of random noises. To evaluate whether the synthetic data own the similarity and diversity with the real data, the classification accuracy index is adopted in this article. A public EMG dataset (that is, ISR Myo-I) for hand motion recognition is used to prove the usability of the proposed method. The experimental results show that adding synthetic data to the training data has little effect on the classification performance, indicating the similarity between real data and synthetic data. Moreover, it is also noted that the average accuracy (five classes) is slightly increased by 1%–2% for support vector machine (SVM) and random forest (RF), respectively, with additional synthetic data for training. Although the improvement is not statistically significant, it implies that the generated data by DCGAN own its new characteristics, and it is possible to enrich the diversity of the training dataset. In addition, cross-validation analysis shows that the synthetic samples have large inter-class distance, reflected by higher cross-validation accuracy of pure synthetic sample classification. Furthermore, this article also demonstrates that histogram equalization can significantly improve the performance of EMG-based hand motion recognition.

## 1 Introduction

Bio-signal analysis plays a crucial role in disease diagnosis, rehabilitation medicine, and even human-machine interaction. Since the discovery of bioelectricity two hundred years ago, scientists have begun to understand the human body’s movement response on a deeper level. 24-h ECG monitoring helps physicians better diagnose patients’ diseases and intervene in treatment; humans have already been able to use electrical signals from the brain for more precise anesthesia; EMG describes the neuromuscular pathology, and it has been used as muscle computer interfaces (MCIs) to interact with extra devices (Chae et al., 2011; Yang et al., 2020). Surface electromyography (sEMG) is also increasingly well-studied, and pattern recognition has been well-used in this regard as well (Jiang et al., 2019; Cheng et al., 2021; Liu et al., 2022). Cheng et al. (2020) have proposed an sEMG system for 2D visualization. With the development of machine learning, object detection technology (Huang et al., 2022), and their progressive application in biology, large-scale bio-signal datasets are mandatory. Picking up the US medical community as an example, the percentage of providers using electronic health records (EHR) increased from 9.4% in 2008 to 83.8% in 2015 (Henry et al., 2016). However, it is difficult to obtain immense bio-signal datasets due to the following reasons. First, the process of extracting bio-signal data is quite cumbersome and requires a strict ethical approval process (Alexiou et al., 2013). Second, both equipment and volunteers have high requirements for high-precision bio-signal acquisition. Therefore, building bio-signal datasets is an extremely costly affair. In addition, the use of bio-signal datasets may pose the problem of privacy breach, even if the dataset is de-labeled, the hidden data can still be recovered by linking to other identifiable datasets (El Emam et al., 2011; Erlich and Narayanan 2014). In this case, the use of qualified synthetic data instead of real data represents a great advantage. It greatly simplifies the process of obtaining data and protects the privacy of testers as much as possible.

Generative adversarial networks (GANs) have demonstrated their power in data enhancement and image processing (Han et al., 2018; Cherian and Sullivan 2019), which is proposed by Goodfellow et al. (2014). To obtain better synthesis results, a large number of GAN models are proposed. For example, conditional GAN (Mirza and Osindero 2014) adds additional conditional information to the generator and discriminator; Deep Convolutional GAN (DCGAN) introduces Convolutional Neural Networks (CNN) (Radford et al., 2015); Wasserstein GAN (WGAN) uses the Wasserstein distance for Jensen–Shannon (JS) divergence (Arjovsky et al., 2017), and so on. In order to better process time series signals, some scholars have devoted themselves to optimizing the network structure, modifying the optimizer and convolution layer used, so that GAN can be better used for time series signal generation (Jordon et al., 2018; Yoon et al., 2019). Zhang et al. (2021) and Tian (2021) use wavelet transformation and Fourier transformation to process data in time series signals.

GANs have been applied in the generation of images and bio-signals. Zhong and Zhao (2021) use the combination of short-time Fourier transformation and GAN to extract fetal ECG; Hartmann et al. (2018) study the application of WGAN in electroencephalogram (EEG); Hazra and Byun (2020) used bidirectional grid long- and short-term memory for the generator network and constructed SynsigGAN for synthesizing various types of bio-signals. The remarkable ability of GAN makes it competent for other related applications. Khan et al. (2021) propose an adversarial Gaussian denoiser network that enables good Gaussian denoising of images. Utilizing DCGAN, the accuracy of grayscale ear image recognition is greatly improved (Khaldi and Benzaoui, 2021; Khaldi et al., 2021). Fang et al. (2018) utilize DCGAN to generate samples and train in an image recognition model, and achieved satisfactory classification performance in the radar profile as the dataset for 4 categories. Fang et al. (2019) proposed a new gesture recognition algorithm based on the CNN and DCGAN, and it becomes less susceptible to illumination and background interference.

In recent three years, GAN has been used to process the EMG signal. Chen et al. (2021) use a GAN-based separation framework to separate the class-related EMG features for the detection of trunk compensatory patterns in stroke patients. Anicet Zanini and Luna Colombini (2020) take DCGAN and neural style transfer to simulate each patient’s EMG tremor pattern with different frequencies and amplitudes under different sets of movements. Hu et al. (2019) propose a two-step pipeline classification solution based on adversarial learning, achieving better gesture classification accuracy for both sparse multi-channel sEMG database and the high-density sEMG database. These studies address bio-signals as one-dimensional time series signals, and thus only one-dimensional convolutional layers are adopted. The contributions of this study are as follows:

• This article proposes a method for generating synthetic data. With a small amount of real EMG data, the proposed method can transfer noise into synthetic EMG data by DCGAN, and the generated data can enrich the dataset to train classifiers more sufficiently.
• This article does not rely on any traditional EMG feature, and only transfers the raw EMG data signal to images through matrix formalization, normalization, and histogram equalization. During the classification part, all pixel values will be changed to vectors.
• Our experimental results prove that the synthetic data are similar to the real EMG data; the synthetic samples have a larger inter-class distance in comparison with the real samples; image histogram equalization can significantly enhance the performance of the proposed method.

## 2 Methods

**FIGURE 1:**
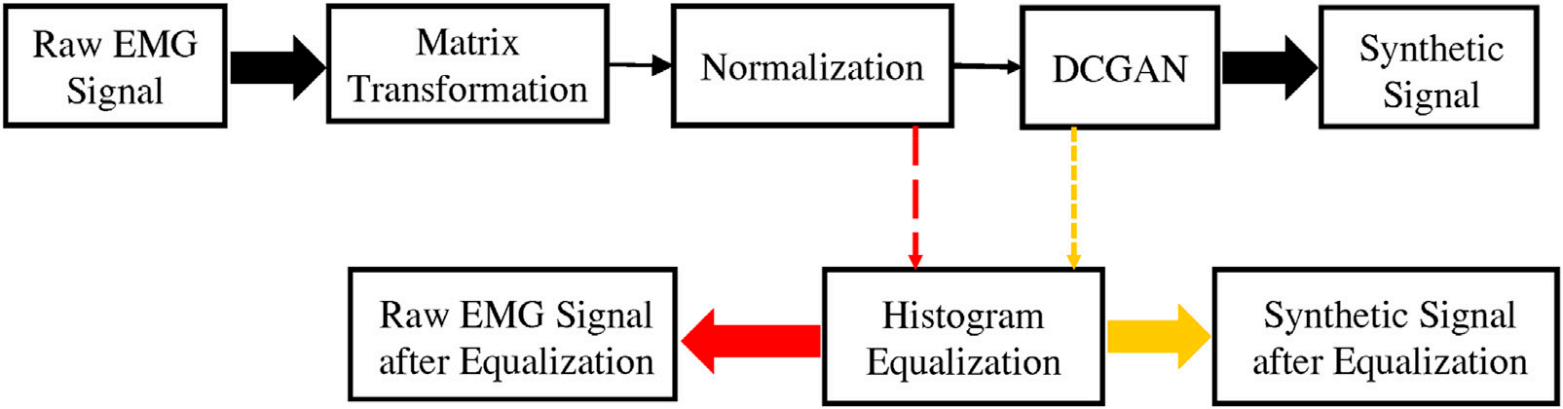
It briefly describes the methods used in this article including matrix transformation, normalization, histogram equalization, and DCGAN.

Figure 1 shows the general flow of the proposed method in this article. The raw EMG data after matrix transformation and normalization will be converted into image signals. After the signal is equalized, the R-E dataset in Section 3.3 can be obtained. After the raw image dataset is processed by the DCGAN, a synthetic dataset can be obtained. It passes the image equalization algorithm and will get the F-E dataset in Section 3.3.

### 2.1 Matrix Transformation and Normalization

The data preprocessing consists of matrix transformation and normalization. The dimensionality of the matrix transformation is in line with the experimental dataset. The specific dataset will be introduced in Section 2.5.1. The matrix transformation reshapes the size of matrices from 16 by 256 to 64 by 64.

$$\begin{array}{c} B=T\left(A\right) \end{array}$$ (1)

where **A** is the original matrix, and **B** is the converted matrix. In detail,

$$\begin{array}{c} A=\left[\begin{array}{ccc} {\text{a}}_{1,1} & \cdots & {\text{a}}_{1,256}\\ \vdots & \ddots & \vdots \\ {\text{a}}_{16,1} & \cdots & {\text{a}}_{16,256} \end{array}\right] \end{array}$$ (2)

where ${\text{a}}_{i,j}$ is the EMG value, *i* and *j* indicate the channel and time point, respectively. After transformation,

$$\begin{array}{c} B=\left[\begin{array}{ccc} {\text{a}}_{1,1} & \cdots & {\text{a}}_{1,64}\\ \vdots & \ddots & \vdots \\ {\text{a}}_{16,193} & \cdots & {\text{a}}_{16,256} \end{array}\right] \end{array}$$ (3)

Normalization makes each value in each matrix range between 0 and 255 so that each matrix can be displayed as a grayscale image. The normalization is

$$\begin{array}{c} b_{i,j}=255\frac{a_{i,j}-a_{min}}{a_{max}-a_{min}} \end{array}$$ (4)

where

$$\begin{array}{c} a_{min}=\min\left(A\right) \end{array}$$ (5)

and

$$\begin{array}{c} a_{max}=\max\left(A\right) \end{array}$$ (6)

where min(A) and max(A) indicate finding the maximum and minimum values in matrix **A**.

After preprocessing, an EMG sample becomes a 64 by 64 grayscale image. The reason for acquiring a grayscale image is to facilitate the use of established computer vision and image processing techniques, such as 2D convolutional networks.

### 2.2 Histogram Equalization

Since this article does not use methods, such as feature extraction, a large amount of noise exists in both real and synthetic images. Therefore, this article uses the histogram equalization method to perform image enhancement processing on the generated images.

If the probability density function of the known random variable *r* is $p_{r}\left(r\right)$ , and the random variable *s* is a function of *r*, that is, *s=T*(*r*), the probability density of *s* is $p_{s}\left(s\right)$ . So $p_{s}\left(s\right)$ can be calculated from $p_{r}\left(r\right)$ :

$$\begin{array}{c} p_{s}\left(s\right)=p_{r}\left(r\right)\left|\frac{dr}{ds}\right| \end{array}$$ (7)

Combined with another important transformation in image processing:

$$\begin{array}{c} s=T\left(r\right)=\left(L-1\right)\int_{0}^{r}p_{r}\left(w\right)dw \end{array}$$ (8)

where *L* is the gray level of the image and *w* is the pseudo-integral variable. Then ask for

$$\begin{array}{c} \frac{ds}{dt}=\frac{dT\left(r\right)}{dt}=\left(L-1\right)\frac{d}{dr}\left[\overset{r}{\underset{0}{\int }}p_{r}\left(w\right)dw\right]=\left(L-1\right)p_{r}\left(r\right) \end{array}$$ (9)

Then bring in

$$\begin{array}{c} p_{s}\left(s\right)=p_{r}\left(r\right)\left|\frac{dr}{ds}\right|=p_{r}\left(r\right)\left|\frac{1}{\left(L-1\right)p_{r}\left(r\right)}\right|=\frac{1}{\left(L-1\right)} \end{array}$$ (10)

The histogram equalization formula can be obtained:

$$\begin{array}{c} s_{k}=T\left(r_{k}\right)=\left(L-1\right)\overset{k}{\underset{j=0}{\sum }}p_{r}\left(r_{j}\right),k=0,1,\ldots ,L-1 \end{array}$$ (11)

where $r_{k}$ is the grayscale of the input image, $s_{k}$ is the grayscale of the output image. Moreover, in order to better judge the data enhancement method proposed in this article, the real pictures are also equalized to reduce the interference with the experimental results.

**FIGURE 2:**
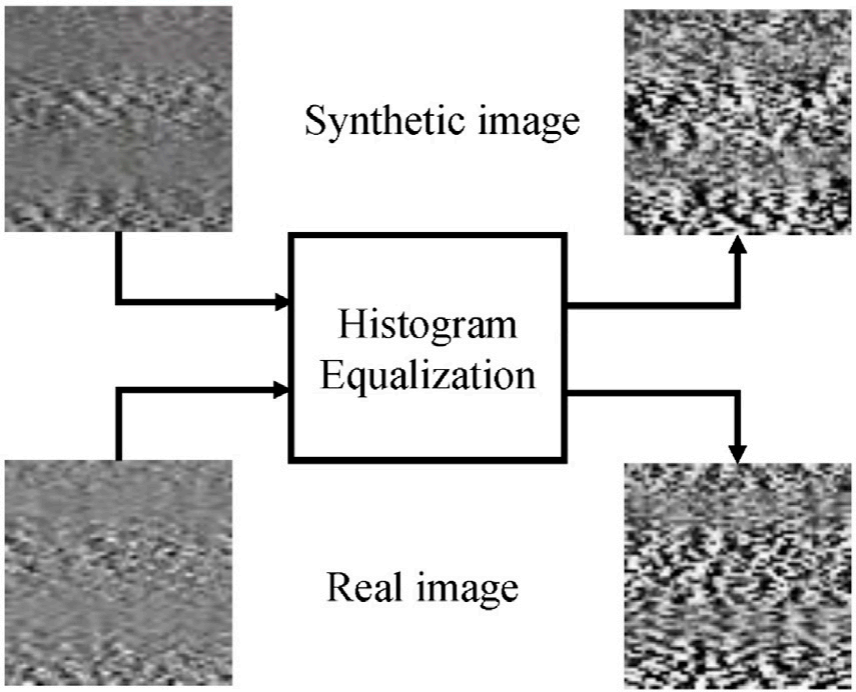
It shows the grayscale images before and after equalization. The top two images are synthetic images, and the bottom two are real ones.

Figure 2 shows the effect of equalization, and it can be seen that the acutance is enhanced for both real and synthetic images.

### 2.3 Framework for Generative Adversarial Networks

In the initial GAN, two models are simultaneously trained in the whole framework: one is the generative model ***G*** that obtains the data distribution, and the other is the discriminative model ***D*** that determines whether the sample comes from the training data. The training procedure for ***G*** is to maximize the probability that ***D*** is wrong. ***G*** receives a random noise ***z*** and generates a picture through this noise, denoted as ***G(z)***. ***D*** is used to determine whether a picture is “real” or not. The input is an image, it's named x. The output ***D(x)*** indicates whether x is a real image. In the training process, the goal of generating network ***G*** is to try to generate real pictures to deceive the discrimination network ***D***. The optimal objective of the GAN can be expressed as V(D,G) :

$$\begin{array}{c} \underset{\text{G}}{\text{min}}\;\underset{\text{D}}{\text{max}}V\left(D,G\right){=}_{x∼p_{data}\left(x\right)}\left[logD\left(x\right)\right]{+}_{z∼p_{z}\left(z\right)}\left[log\left(1-D\left(G\left(x\right)\right)\right)\right] \end{array}$$ (12)

where $p_{data}\left(x\right)$ is the distribution of real data, $p_{z}\left(z\right)$ is the distribution of noise, and is mathematical expectation. The calculation of the optimal discriminant network can be proved as follows:

$$\begin{array}{c} V\left(G,D\right)=\underset{x}{\int }p_{data}\left(x\right)\text{log}\left(D\left(x\right)\right)+p_{g}\left(x\right)\text{log}\left(1-D\left(x\right)\right)dx \end{array}$$ (13)

where $p_{g}\left(x\right)$ is the x distribution generated by z. In order to simplify the writing of the equation, it may be assumed that

$$\begin{array}{c} p_{data}\left(x\right)=a,p_{g}\left(x\right)=b,D\left(x\right)=y \end{array}$$ (14)

Putting the assumptions into Eq. 8, the integral function f(y) can be obtained as

$$\begin{array}{c} f\left(y\right)=alog\left(y\right)+blog\left(1-y\right) \end{array}$$ (15)

and then, according to the first and second derivatives of f(y) , can get y=a/(a+b) is the extreme point, the function takes the maximum value at this time.

$$\begin{array}{c} V\left(G,D\right)=\int_{x}f\left(y\right)dx\leq \int_{x}\underset{y}{\max}f\left(y\right)dx \end{array}$$ (16)

The calculation of the optimal generative network is as follows:

$$\begin{array}{l} C\left(G\right)=\underset{x}{\int }p_{data}\left(x\right)log\left(D^{G}\left(G\right)\right)+p_{g}\left(x\right)log\left(1-D_{G}^{*}\left(x\right)\right)\\ \begin{array}{c} =-log4+\underset{x}{\int }alog\frac{a}{\frac{a+b}{2}}+alog\frac{b}{\frac{a+b}{2}} \end{array} \end{array}$$ (17)

where $D_{G}^{*}$ is the optimal generator. By adding numerator 2, two Kullback–Leibler (*KL*) divergences are constructed, and the *KL* divergence is greater than or equal to 0. And, assuming that there are two distributions ***A*** and ***B***, and the average distribution of these two distributions $C=\frac{A+B}{2}$ , then the *JS* divergence between them is the *KL* divergence of ***A*** and ***C*** and the *KL* divergence of ***B*** and ***C*** One-half of the *KL* divergence, as follows:

$$\begin{array}{c} JSD\left(A|\text{|}B\right)=\frac{1}{2}KL\left(A|\text{|}C\right)+\frac{1}{2}KL\left(B|\text{|}C\right) \end{array}$$ (18)

Provable

$$\begin{array}{l} C\left(G\right)=-log4+KL\left[a||\frac{a+b}{2}\right]+KL[a||\frac{a+b}{2}\\ \begin{array}{c} =-log4+2JS\left(a||b\right) \end{array} \end{array}$$ (19)

Therefore, the GAN is solvable, and the solution of the optimal generator converges to 1/2. However, the original GAN suffers from problems such as unstable training and easy mode collapse.

**FIGURE 3:**
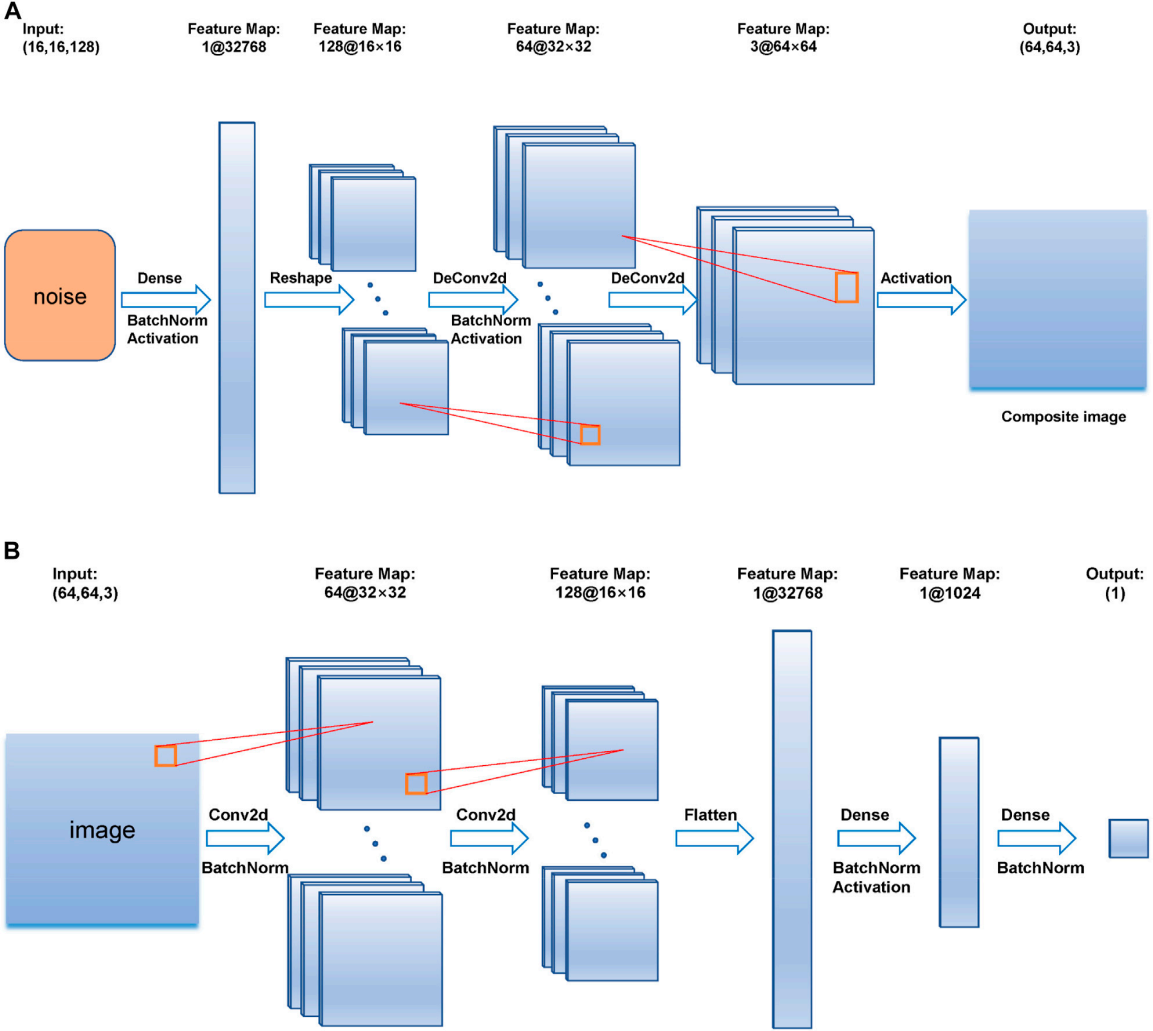
**(A)** is the construction of the generator, **(B)** is the construction of the discriminator.

This article chooses DCGAN as the network to generate synthetic EMG signals. DCGAN is sourced from the original GAN, but uses convolution and deconvolution instead of pooling layers, and uses the Tanh activation function instead of the output layer of the generator, etc. The specific network framework is shown in Figure 3. The network model has 9 layers in the generative network, and 10 layers in the discriminant network. In the generation network, the first layer is a fully connected layer with an input size is 16 by 16 by 128; the second and seventh layers are Batch Normalization (BN) layers, making the optimization space smoother; the third, sixth, and ninth layers are activation layers with tanh activation function; the fourth layer is a reshape layer; the fifth and eighth layers are deconvolution layers, with kernel size and stride to 5 and 2, respectively. The output is a 64 by 64 RGB image. In the first layer of the discriminant network, the input size is 64 by 64 by 3. To follow the input demand of DCGAN, the matrix A is copied 3 times to the shape of [A, A, A]. This layer is similar to the third layer of the convolutional layer, which sets the kernel size and step size to 5 and 2, respectively. The second, fourth, and seventh layers are all BN layers. The fifth layer is a flattened layer, which converts the multi-dimensional output into one dimension. The sixth layer is a fully connected layer with an output length of 1024. The eighth layer is an activation layer with an activation function of tanh. The ninth layer is a fully connected layer. The last layer is a sigmoid-based activation layer.

### 2.4 Hyperparameter Determination

**FIGURE 4:**
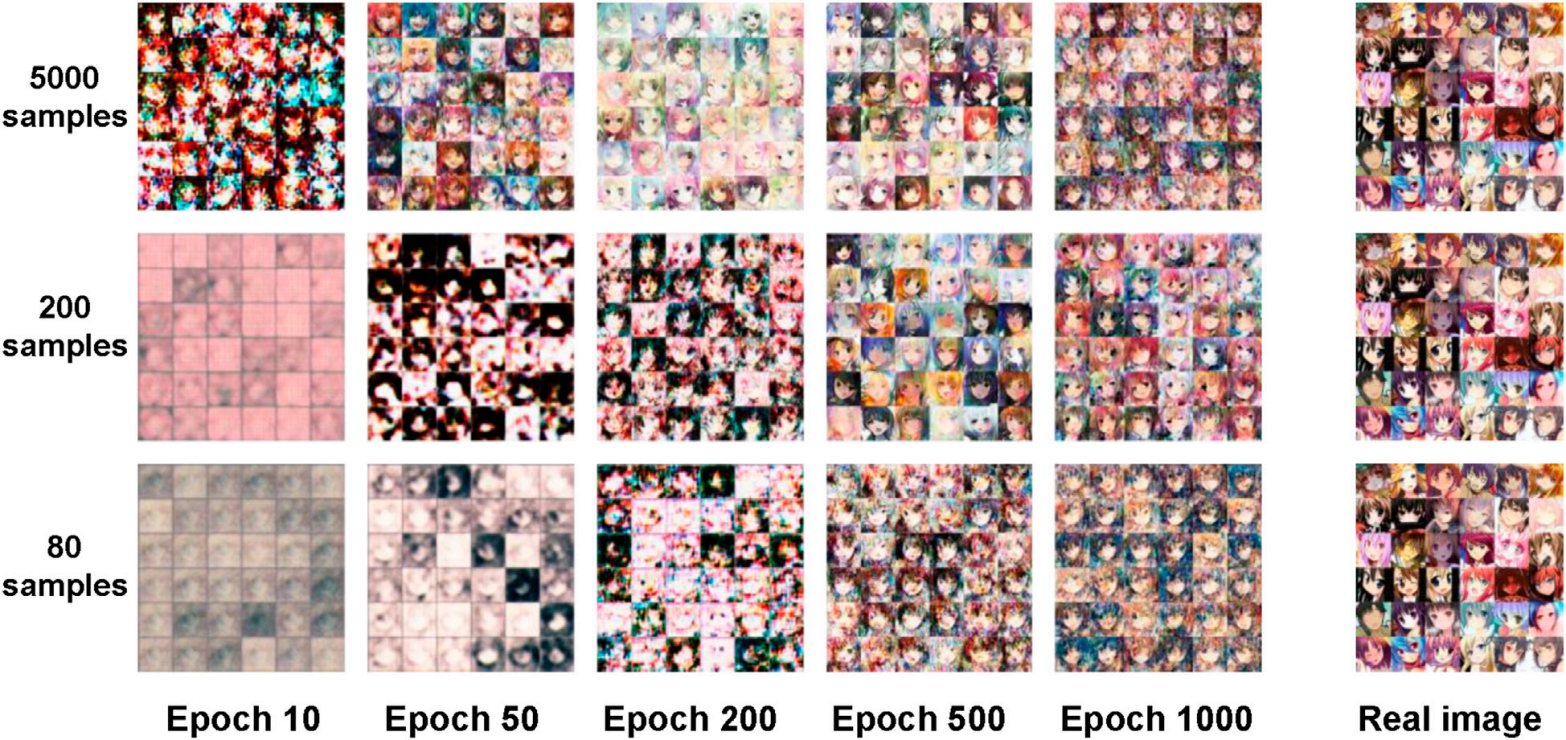
The first, second, and third rows show the synthetic images with sample numbers of 5000, 200, and 80, respectively. The first five columns show the synthetic images at different epochs. The last column shows the real images.

Feasibility and stability are important metrics for DCGAN. 60,000 RGB images with pixels of 64 by 64 by 3 in the toy dataset are introduced to test the DCGAN model, and the batch size is set to 128 to observe the output under 1000 epochs. The toy dataset is used to test the generative effect of the constructed GAN and to help us experiment with the choice of hyperparameter. Since the basis of this article is still to take the advantage of GAN for image generation, the toy dataset is also an image dataset. In the stage of network construction, a dataset that can be visually inspected is required to detect whether the most basic functions of the network are feasible. Therefore, this article chooses the avatar dataset from Kaggle as the toy dataset for evaluation. To verify whether the GAN functions well on a small training dataset, this article divides the toy dataset into minor ones. The result is compared with that of the previous GAN for large sample training, which helps us to roughly determine the setting of hyperparameter. It allows us to quickly judge whether the constructed DCGAN model is stable. After confirming that the network model has the initial ability to generate images, this article reduces the number of samples to 200 and 80 to train the DCGAN for small-size data evaluation. As shown in Figure 4, the quality of the synthesized images decreases when the number of samples is reduced. But it can still provide acceptable performance as long as the number of epochs is large enough. Therefore, it can be considered that the network constructed is stable and feasible. Based on preliminary experiments on the toy dataset, this article sets batch size to 32 and epoch to 1000.

**FIGURE 5:**
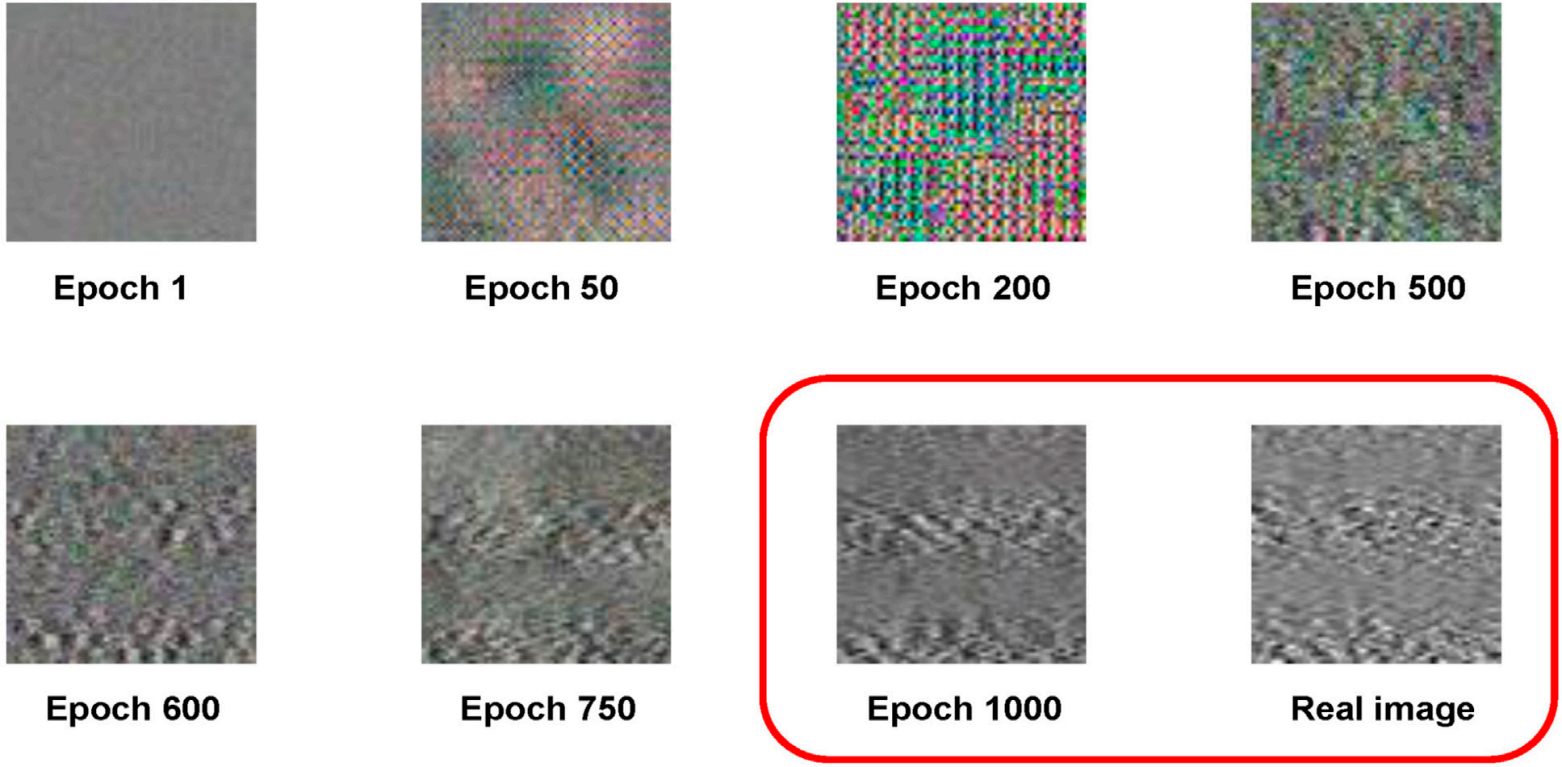
It shows the EMG synthetic images trained by 80 samples at different epochs and compares synthetic images at epoch 1000 with the real image.

Figure 5 shows the synthetic EMG images generated by DCGAN at different epochs, in which the DCGAN is trained by the EMG data of a gesture. It can be found that with the increase of epoch, the quality of the image improves. In comparison with the real EMG image, the synthetic one shows a very similar appearance at 1000 epochs. Thus, the last 100 synthetic images at 901–1000 epochs are selected as the synthetic dataset.

### 2.5 Evaluation

#### 2.5.1 Electromyography Dataset

The dataset in this article is the target of one with sEMG signals, which is initially proposed by Fang et al. (2021). It is measured with a custom-built acquisition system. The system consists of 16 bipolar surface EMG channels. Each channel of the EMG signal is filtered by a 50 Hz powerline filter, followed by an analog-to-digital converter with a sampling frequency of 1 kHz. In this dataset, 16 channels of EMG signals are acquired from the forearm when subjects are performing 13 hand gestures. Six subjects are involved in the procedure of data acquisition. For each subject, data are collected for 10 days, and twice each day in the morning and afternoon. After data segmentation and labeling, each gesture contains 100 samples, and each sample has 4,096 values. The 4,096 values consist of 16 channels of 256 sampling points. In our experiments, part of the dataset is chosen to evaluate the proposed method, containing a total of 10 gestures in 4 days. More detailed information about the experimental data will be given in Section 2.5.2.

**FIGURE 6:**
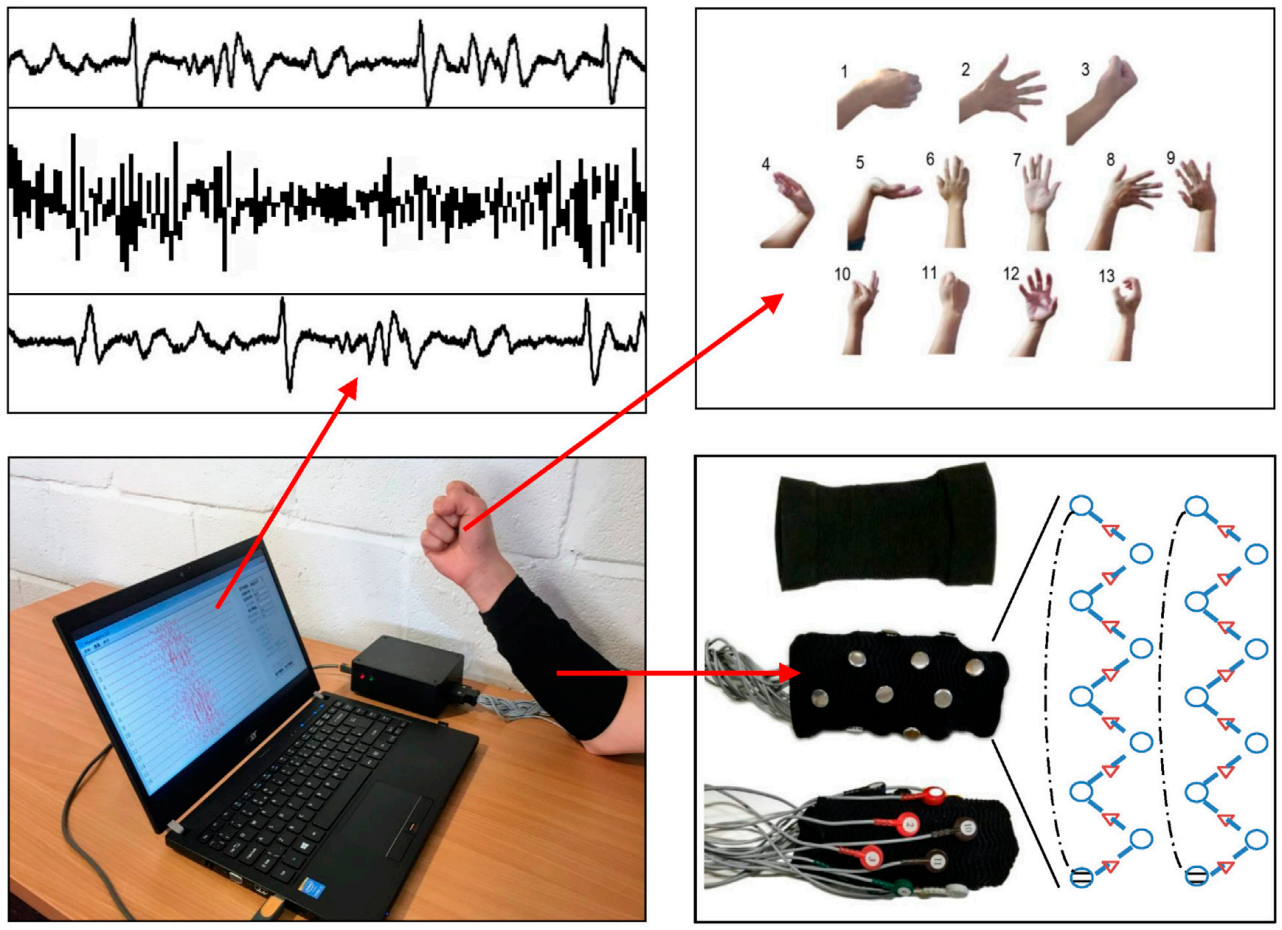
It is a conceptual diagram of the experimental setup and data collection scenario. It describes the process of collecting EMG signals of 13 gestures under 16 channels.

The EMG signal acquisition scenario is illustrated in Figure 6, where 13 specific hand gestures are demonstrated. The picture in the upper left shows the EMG signal displayed on the screen. The remaining parts show the instruments and equipment used in the experiment, such as the EMG amplifier and the 16-channel sensory sleeve.

#### 2.5.2 Classification

The designed DCGAN is tested on part of the ISRMyo-I dataset. The selected sub datasets are referred to as S1D1, S3D1, S5D1, S1D2, S3D2, S5D2, S2D5, S4D5, S6D5, S2D3, S4D3, and S6D3. S1D1 indicates the EMG data recorded from the first day of the first subject. The first six sub datasets are selected from the morning session with gesture labels 1, 3, 5, 9, and 10. The first six sub datasets are selected from the afternoon session with gesture label 2, 4, 6, 7, and 8.

For the training of DCGAN, one of the first 80 images out of 100 images is selected, and a separate model is trained for each gesture. The synthetic images are generated along with the training procedure, and thus 1,000 fake images are generated after 1,000 epochs. In this article the last 100 synthetic images are included in the synthetic dataset for further testing.

This article uses the Weka (University of Waikato, New Zealand) packing classifiers (SVM and RF) with the default parameters to evaluate the experimental results. This article reconverts 64 by 64 image data into 1 by 4,096 vectors. For better classification, all the values in the vector are normalized to the range between 0 and 1. Adding the label to the vector, an EMG sample contains 4,097 columns, which becomes the input of Weka-based classifiers.

This article conducts two experiments to evaluate the effect of DCGAN: similarity analysis and cross-validation analysis. In the similarity analysis, 80% of the real data (80 samples per gesture in each sub dataset), synthetic data (100 samples per gesture in each sub dataset), and mixed data (containing 80% of the real data and synthetic data) are used to train SVM and RF classifiers, and the rest 20% of the real data (20 samples per gesture in each sub dataset) are used for testing. The classification accuracy indicates whether the synthetic data is similar to the real data and whether it can contribute to the enrichment of the dataset. If the classifier trained by synthetic data can classify real data, it somewhat indicates synthetic data has a certain similarity with the real data. If the classifiers trained by a mixture dataset can achieve higher classification accuracy, it implies that synthetic data can enhance the data in a good manner for classifier training. In the cross-validation analysis, a 10-fold cross-validation analysis is performed on each dataset to judge the inter-class and intra-class distance for each gesture. Higher classification accuracy can somewhat indicate larger inter-class and less intra-class distance. In the experiments, real data (**R**), fake data (**F**), real data after equalization (**R-E**), fake data after equalization (**F-E**), the mixture of fake data and real dataset (**FR**), the mixture of fake data and real data after equalization (**FR-E**) are separately tested under 10-fold cross-validation. For each subject, the **R** dataset contains 500 samples (5 gestures, and 100 samples per gesture), and the **F** dataset contains 500 samples as well, but these samples are generated by DCGAN.

## 3 Results

### 3.1 Similarity Analysis

Table 1shows the similarity analysis results by the index of classification accuracy. It can be found that when the classifier is only trained by synthetic data, the average accuracy decreases by 4.72%, from 59.19% to 54.47%. However, after mixing the real data with synthetic data, the average accuracy increases to 61.85%, which is 2.66% higher than that of real data trained classifiers. It can be found that the overall accuracy is not high, which is probably because all classifiers are trained by 4,096-dimension samples which are directed converted from images. It is also found that RF outperforms SVM in all cases in our experiments.

**TABLE 1:** The classification accuracy is obtained by SVM and RF classifiers, where 2-, 3, and 5-class gesture classification problems are considered. For all the tests, 20 real samples in each gesture are used to test the classifiers trained by real data (RR), synthetic data (FR), and both real data and synthetic data (MR).

Classification	RR (%)	FR (%)	MR (%)	Promotion (%)
SVM 2 class	63.93	60.00	65.36	1.43
SVM 3 class	46.25	45.62	49.17	2.92
SVM 5 class	32.50	29.25	33.50	1.00
RF 2 class	86.94	83.06	90.28	3.34
RF 3 class	71.67	67.61	76.90	5.23
RF 5 class	53.86	41.29	55.86	2.00
Average	59.19	54.47	61.85	2.66

### 3.2 Cross-Validation Analysis

**FIGURE 7:**
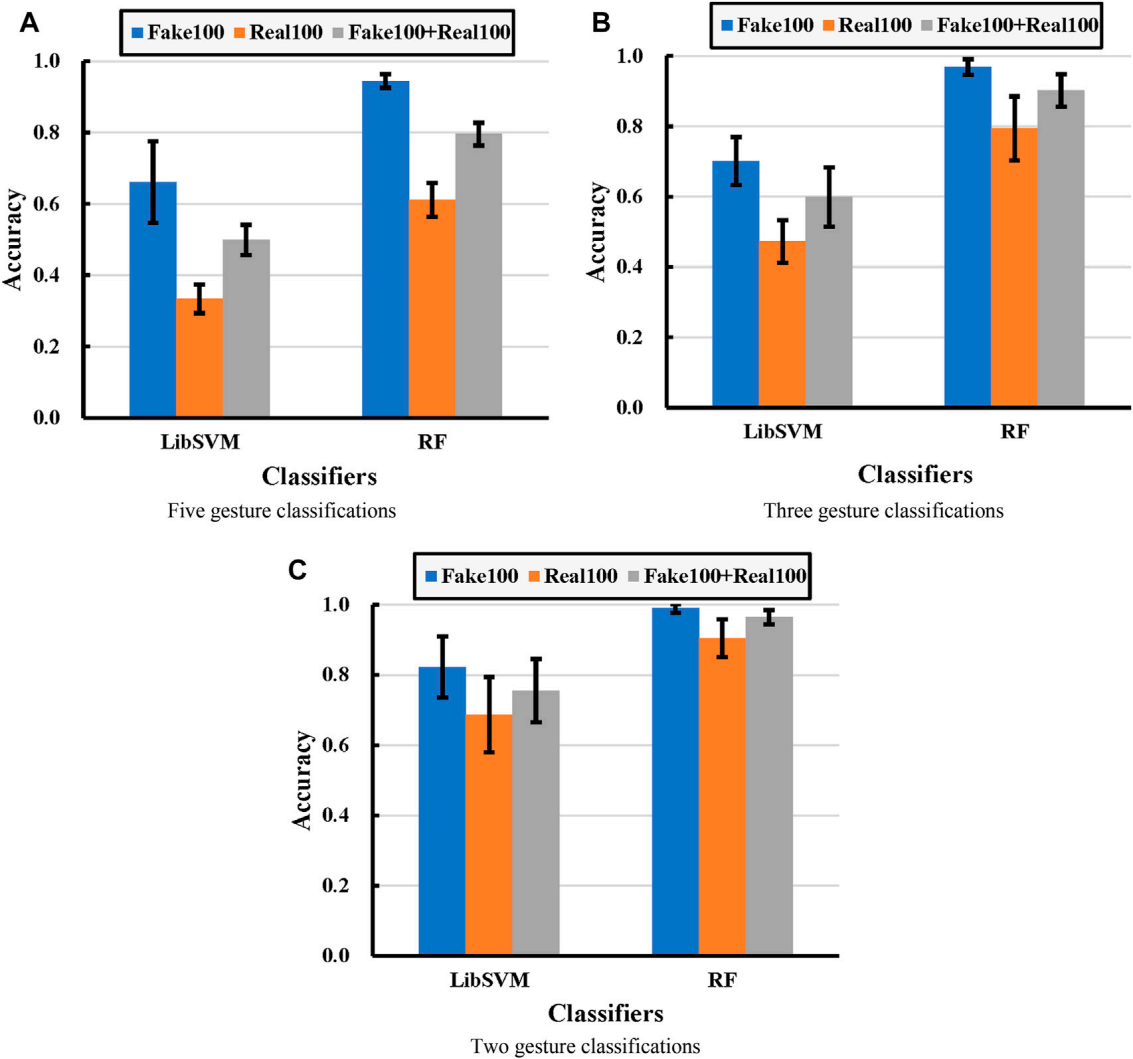
Experimental comparisons under different classifications, in which **(A)**, **(B)**, and **(C)** correspond to five-, three-, and two-class classification problems, respectively. Each bar represents the classification accuracy, and the error bar represents the corresponding standard deviation. Real100 means cross-validation with 100 real images for each gesture. Fake 100 represents cross-validation with 100 fake images for each gesture. Fake100+Real100 means cross-validation with 100 fake images and 100 real images mixed for each gesture.

From the experimental results in Figure 7, it can be seen that the classification accuracy obtained from the synthetic dataset (Fake 100) is the highest in comparison with the other two, and the classification accuracy obtained from the real dataset (Real 100) is the lowest. After mixing the real data with the fake data (Fake 100 + Real 100), it can also be found that the accuracy obtained is lower than the Fake 100 test, but higher than that of the Real 100 test.

For SVM to classify five gestures, the average accuracy obtained from real data, fake data, and mixed data are 33.32%, 66.07%, and 49.86%, respectively. For SVM to classify three gestures, the average accuracy obtained from real data, fake data, and mixed data are 47.28%, 70.17%, and 59.90%, respectively. For SVM to classify three gestures, the average accuracy obtained from real data, fake data, and mixed data are 68.67%, 82.21%, and 75.52%, respectively. For RF to classify five gestures, the average accuracy obtained from real data, fake data, and mixed data are 61.10%, 94.47%, and 79.55%, respectively. For RF to classify three gestures, the average accuracy obtained from real data, fake data, and mixed data are 79.44%, 96.86%, and 96.86%, respectively. For RF to classify two gestures, the average accuracy obtained from real data, fake data, and mixed data are 90.46%, 96.44%, and 98.92%, respectively.

Overall, the data exhibited similar enhancement curves across all experiments. It implies that the synthetic data have a larger inter-class and smaller intra-class under-distance than the real data. That is the reason why the cross-classification of synthetic data and mixed data achieves much higher classification accuracy than the real dataset.

### 3.3 Histogram Equalization Enhancement Analysis

**FIGURE 8:**
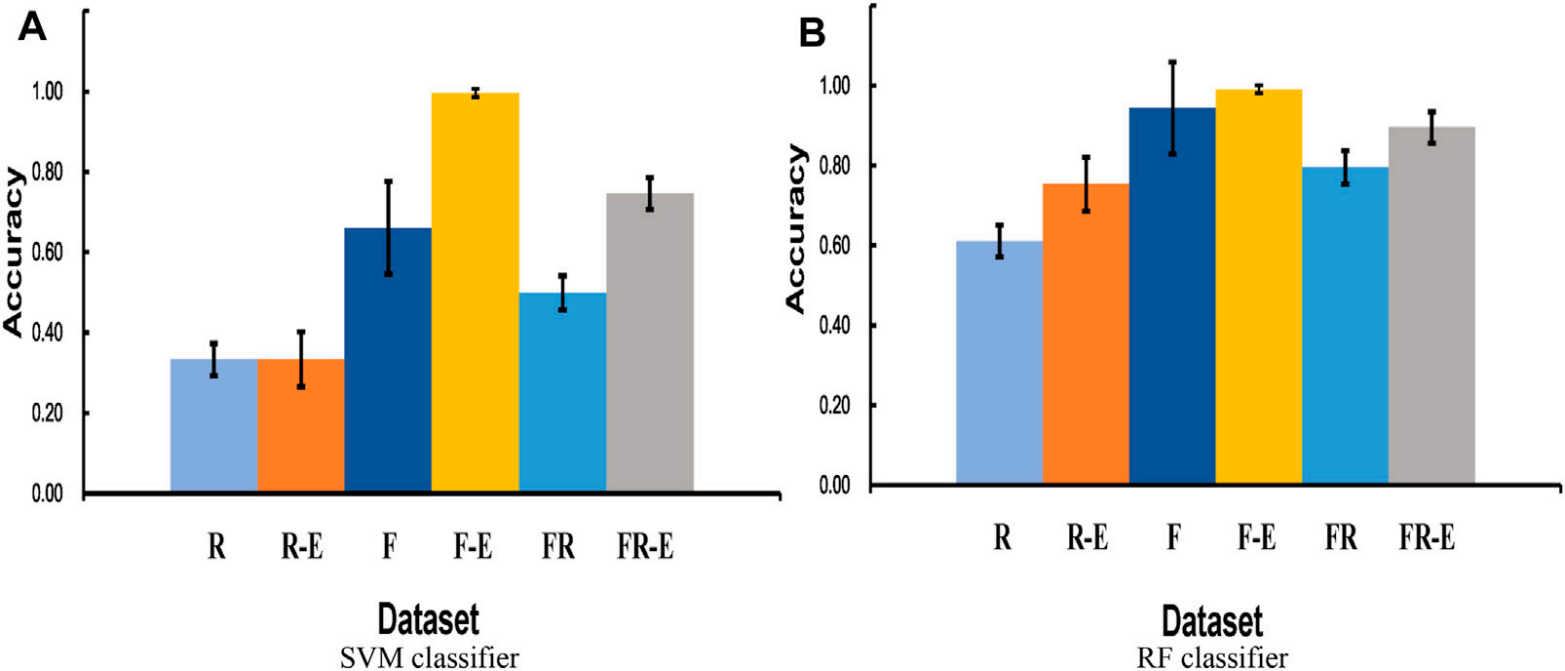
**(A)** compares the cross-validation accuracy across different datasets by SVM-based classification, and **(B)** compares the cross-validation accuracy across different datasets by RF-based classification. **R** and **F** represent that only real data and fake data are used, respectively. **R-E** and **F-E** represent the real and fake datasets after equalization, respectively. **FR** represents a mixture dataset with equal amounts of real data and fake data. **FR-E** represents a mixture of equalized datasets containing both real and fake data.

Further experimental results are conducted on the effect of histogram equalization on both similarity test and cross-validation test. As seen in Figure 8 **(**similarity tests**)**, the data after equalization achieves better classification accuracy in both SVM and RF classifiers. SVM classifier seems to be insensitive to histogram equalization for the real dataset (**R**), and the classification accuracy after histogram equalization for **F** and **FR** datasets is substantially improved by 33.60% and 24.78%, respectively. Since the original classification accuracy of the RF classifier is relatively higher, the improvement rate of equalization is not that significant. But the improvement is still evident in Figure 8B.

**FIGURE 9:**
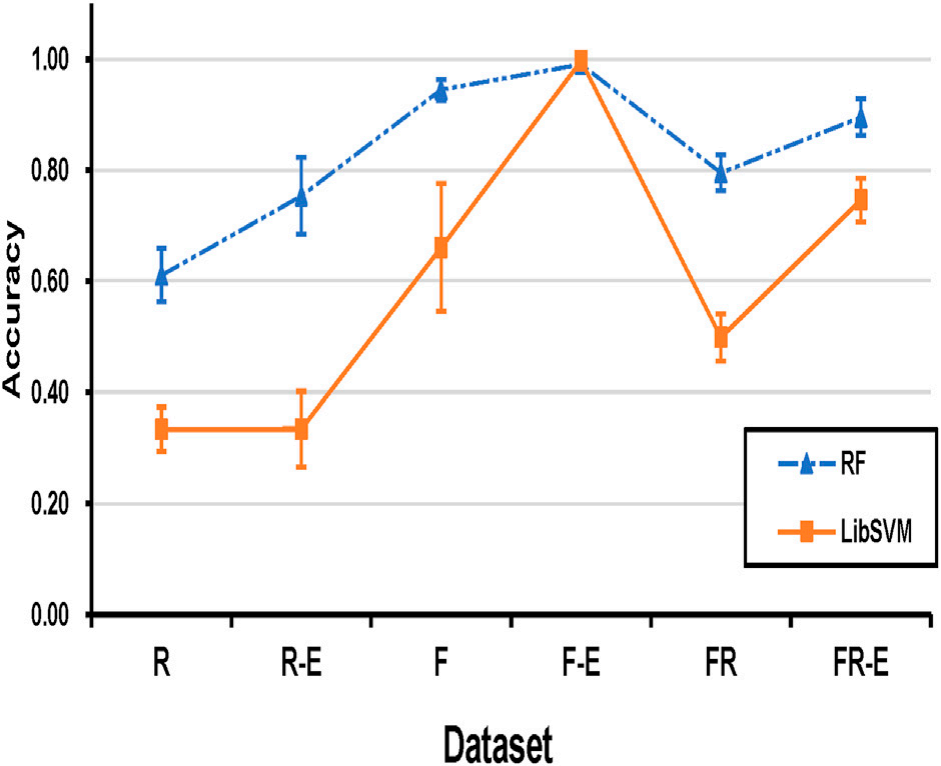
10-fold cross-validation accuracy with different datasets, including real data **(R)**, fake data **(F)**, real data after equalization **(R-E)**, fake data after equalization **(F-E)**, the mixture of fake data and real data **(FR)**, the mixture of fake data and real data after equalization **(FR-E)**.

Figure 9 (cross-validation test) clearly shows that the classification accuracy of the **F** dataset is much higher than that of the **R** dataset, and the classification accuracy of the **FR** dataset is slightly higher than their average. The equalization approach is successful for almost all datasets and classifiers. It makes the five-class accuracy of the FR dataset finally reach 74.63% and 89.52% in the SVM and RF classifiers, respectively.

## 4 Discussion

This article investigates a DCGAN-based method for multi-channel EMG signal enhancement and validates the results using two classifiers packaged in Weka. The classification ability of the two selected classifiers, SVM and RF, has been corroborated in several ways. Among them, RF is more suitable for the classification of high-dimensional data, so in each group of experiments, the classification accuracy of RF is better than SVM. However, since the dataset in this experiment does not take feature extraction and classifier parameter adjustment, the overall classification accuracy is not high.

In the similarity test, the mixed dataset containing synthetic data maintains comparable classification accuracy to the real dataset. The experiment using synthetic data for training and real data for tests, does not result in a significant accuracy drop. These results prove that the synthetic dataset has a certain similarity with the real dataset, which can be effectively enriched. In a cross-validation test, this article finds that synthetic data can provide higher classification accuracy than real data, which implies that the synthetic dataset may have larger inter-class or smaller intra-class distances.

## 5 Conclusion

This article proposes a method to generate synthetic EMG data for hand gesture classification by DCGAN, in which real multi-channel EMG signals are converted to images for DCGAN training, and histogram equalization is adopted in order to process the image for better performance. Our experiment proves that synthetic data can enrich the sample pool to improve the classification accuracy to a certain extent. Histogram equalization can further improve images quality of both real and synthetic images. The reason may be the case that histogram equalization enhances the global contrast of the images, allowing better classification accuracy. In the future, synthetic data will be further tested in deep learning networks, to verify whether the addition of synthetic data can promote deep learning network training. Additional GAN models and image enhancement methods will also be tested to further enhance the quality of synthetic EMG.

## Data Availability

The datasets presented in this study can be found in online repositories. The names of the repository/repositories and accession number(s) can be found at: https://github.com/yinfengfang/ISRMyo-I.git
